# Sociodemographic and Clinical Features in Patients Presented With Accidental and Deliberate Self-Poisoning: A Comparative Study from Lady Reading Hospital Medical Teaching Institution, Peshawar, Pakistan

**DOI:** 10.7759/cureus.10935

**Published:** 2020-10-14

**Authors:** Nasrullah Zadran, Jasvindar Kumar, Asfandyar Ibrar, Abdul Wali Khan, Abat Khan, Muhammad Ishaq, Amber Tahir

**Affiliations:** 1 Internal Medicine, Lady Reading Hospital Medical Teaching Institution, Peshawar, PAK; 2 Internal Medicine, Khyber Teaching Hospital, Peshawar, PAK; 3 Internal Medicine, Hayatabad Medical Complex, Peshawar, PAK; 4 Cardiology, Khyber Teaching Hospital, Peshawar, PAK; 5 Internal Medicine, Dow University of Health Sciences, Karachi, PAK

**Keywords:** clinical, self-poisoning, peshawar, pakistan, asia, tess, aapcc, covid19, poisoning, accidental poisoning

## Abstract

Objectives

To evaluate the sociodemographic and clinical characteristics of patients presented with acute self-poisoning at a tertiary care hospital in Pakistan.

Methods and Patients

A comparative study was conducted at Lady Reading Hospital MTI between May 2018 to May 2019 for a duration of 12 months. All patients diagnosed with acute self-poisoning were included in the study. Patients with inconclusive diagnosis, who were dead prior to the arrival to the hospital, or had an incomplete history of poison exposure were excluded from the study. At the time of arrival to the emergency department, the patient was first stabilized. Patients were grouped into two according to the type of exposure, i.e., accidental self-poisoning and deliberate self-poisoning (DSP). Sociodemographic and clinical characteristics of patients were recorded in a preformed proforma. The data were analyzed using Statistical Package for the Social Sciences (SPSS) Version 26 (IBM Corp., Armonk, NY, USA).

Results

The mortality rate in patients with accidental poisoning was 9.62%, whereas it was 26.28% in DSP patients. Data were stratified according to the mode of poisoning, i.e., accidental vs DSP, and variables were assessed in patients who did not survive. It was found that 60% of patients who died in the accidental group were aged 0-15 years. In contrast, only one patient between aged 0-15 years died in the DSP group and the majority of the deaths occurred in those aged 25.1-35 years (31 [75.6%]).

Conclusions

In conclusion, women more often attempted suicide, whereas males suffered accidental poisoning more frequently. Firstly, we found a female predominance in the DSP group, whereas males were more prevalent in with young children experiencing accidental poisoning. Longer time from ingestion of poison to the arrival is associated with poor patient prognosis.

## Introduction

According to the toxic exposure surveillance system (TESS) of the American Association of Poison Control Centre (AAPCC), more than two million cases of poisonous ingestion have been reported in the year 2018. This amounts to receiving a patient of poison exposure every 15 seconds [1]. Additionally, suicide rates are also increasing at an alarming rate, especially since the declaration of the COVID-19 pandemic [2-3].

The top six poison exposures are pain relievers, cleaning substances, personal care exposures, sedatives, antidepressants, and pesticides [1,4]. Organophosphates are the most common types of insecticides associated with incidental and intentional self-harm [5]. Easy access to these chemicals and medicines and their affordability increase the risk of accidental or deliberate exposure.

Patients with poison exposure may present with a wide array of non-specific symptoms. For instance, a patient with organophosphate exposure may experience neurological symptoms with concomitant respiratory and cardiovascular issues [6]. Delayed diagnosis is one of the most common causes of mortality among these patients. Patient or attendant denial may hinder prompt diagnosis, leading clinicians in the wrong direction.

The sociodemographic and clinical features differ significantly in developing countries compared to developed or high-income countries. Hydrocarbons and pesticides account for the majority of poisoning-related morbidity and mortality in developed or low-income countries like Pakistan. On the other hand, in high-income countries, household or cleaning products or alcohol are common causes of self-poisoning [7]. While children under the age of five years have the highest chance of being poisoned incidentally with household products, intentional drug overdose with paracetamol or antidepressants is most commonly seen with adolescents or young adults [8-9].

Although there have been many studies from Pakistan on the incidence of poisoning, most of these are outdated. Moreover, comparative data on the patient profile between accidental and deliberate self-poisoning (DSP) is limited to draw any reliable conclusions. Therefore, this study evaluated the sociodemographic and clinical characteristics of patients who presented with acute self-poisoning at a tertiary care hospital, Pakistan.

## Materials and methods

A comparative study was conducted at Lady Reading Hospital MTI between May 2018 to May 2019 for 12 months. Ethical approval was obtained from the Institutional Review Board prior to the study. A non-probability convenience sampling technique was applied to enroll patients in the study. Informed verbal as well as written consent was given by the participants.

All patients, irrespective of age and gender, diagnosed with acute self-poisoning were included in the study. Patients with an inconclusive diagnosis of poisoning or those with an incomplete history of poison exposure were excluded from the study. Patients who were suspected to be homicidal were also not included in the study.

At the time of arrival to the emergency department (ED), the patients were first assessed for airway, breathing, circulation, and assurance that they were stable or resuscitated if in an unstable condition. Then the patients were asked about their history of exposure to the poison, the time of ingestion, or inhalation of the toxin. A family history of psychiatric illness was observed. The patients’ past psychiatric history was retrieved. The sociodemographic characteristics of patients were also noted in a predefined pro forma.

Patients were grouped into two according to the type of exposure, that is, accidental self-poisoning and DSP.

The anonymity and privacy of all patients were maintained. Only the principal investigator had access to the original data that was decoded to remove any personal identifiers of patients before statistical analysis.

The questionnaires were filled on paper later entered and analyzed using Statistical Package for the Social Sciences (SPSS Version 25, IBM Corp., Armonk, NY, USA). The majority of the study variables were categorical and were presented using frequency and percentage. The chi-square test and independent t-test were used when suitable. A p-value of <0.05 was set as a cut-off value for statistical significance.

## Results

A significant association was found between the mode of poisoning and time from poison exposure to the arrival at the ED, revealing that 71 (45.5%) of accidental poisoning cases reached the emergency room within 30 minutes, and only 5.7% reached the emergency room after 180 minutes or more. By contrast, time to arrive at the ED exceeded three hours in 7.1% of intentional cases (p < 0.001), as shown in Table 1.

**Table 1:** Characteristics of study participants (n = 312) ED, emergency department

Characteristics	Accidental	Deliberate	p-Value
Age in years			
0-10	60 (38.5%)	2 (1.2%)	<0.001
10.1-15	41 (26.3%)	6 (3.8%)	
15.1-25	32 (20.5%)	14 (8.9%)	
25.1-35	10 (6.4%)	68 (43.5%)	
35.1-45	8 (5.1%)	34 (21.8%)	
>45	5 (3.2%)	32 (20.5%)	
Gender			
Male	94 (60.26%)	46 (29.49%)	<0.001
Female	62 (39.74%)	110 (70.51%)	
Route of administration			
Oral	71 (45.51%)	94 (60.26%)	0.01
Inhalation	85 (54.48%)	62 (39.74%)	
Time from poison ingestion to ED arrival			
<30 minutes	71 (45.5%)	45 (28.8%)	<0.001
31-60 minutes	35 (22.4%)	68 (43.6%)	
61-120 minutes	27 (17.3%)	20 (12.8%)	
120-180 minutes	14 (8.9%)	12 (7.6%)	
>180 minutes	9 (5.7%)	11 (7.1%)	
Psychiatric comorbidities			
Yes	12 (7.69%)	54 (34.62%)	<0.001
No	144 (92.31%)	102 (65.38%)	
History of suicide attempts			
Yes	5 (3.21%)	49 (31.41%)	<0.001
No	151 (96.79%)	107 (68.59%)	
Patient outcome			
Survival	141 (90.38%)	115 (73.72%)	<0.001
Deaths	15 (9.62%)	41 (26.28%)	

The mortality rate in patients with accidental poisoning was 9.62%, whereas it was 26.28% in DSP patients. It was found that 60% percent of patients who died in the accidental group were between 0 to 15 years old. In contrast, out of the 41 mortalities in the DSP group, only one patient was younger than 16 years of age, whereas the remaining were between 25.1 and 35 years of age (31 [75.6%]), as shown in Table 2.

**Table 2:** Comparison of sociodemographic and clinical features of patients with poor outcome after accidental or intentional deliberate self-poisoning. ED, emergency department

Characteristics	Accidental (N = 15)	Intentional (N = 41)
Age in years		
0-15	9 (60%)	1 (2.4%)
15.1-25	2 (13.33%)	6 (14.6%)
25.1-35	3 (20%)	31 (75.6%)
35.1-45	1 (6.67%)	3 (7.3%)
Gender		
Male	8 (53.33%)	17 (41.46%)
Female	7 (46.67%)	24 (58.54%)
Route of administration		
Oral	6 (40%)	23 (56.1%)
Inhalation	9 (60%)	18 (43.9%)
Rectal	-	-
Time from poison ingestion to ED arrival		
<30 minutes	-	-
31-60 minutes	1 (6.67%)	14 (34.1%)
61-120 minutes	5 (33.3%)	8 (19.5%)
120-180 minutes	9 (60%)	9 (21.9%)
>180 minutes	-	10 (24.4%)

## Discussion

Accidental acute poisoning and DSP have been a topic of concern in Pakistan for the past few decades. The incidence of self-poisoning is increasing at an alarming rate worldwide [1,10]. The reason could be the robust industrialization and the easy access to chemicals and drugs such as benzodiazepines, opioids, and over-the-counter drugs such as paracetamol. In this study, we aimed to compare the sociodemographic and clinical characteristics associated with accidental self-poisoning compared to DSP. Additionally, we also assessed the characteristics associated with increased mortality in patients of self-poisoning.

There was a striking difference in gender distribution in both groups, with female dominance in the "DSP group" and male predominance in the "accidental self-poisoning group."

This trend has been noted in previous studies from Pakistan. In a study by Khan and Reza, benzodiazepine self-poisoning was more common in the female population with a 3:2 ratio [11]. In contrast to the present findings, a recently published study by Khan and Ali revealed that more than one-half were male out of the 180 patients of poisoning [12]. However, they found that in patients who attempted suicide by self-poisoning, most were women 81.7%, whereas 71.4% were male. Additionally, males were more common, with a frequency of 23.5% compared to 18.5% of females [12].

We found that the time from poison ingestion to arrival to the ED was significantly associated with the type of self-poisoning. In the case of DSP, the time to present the patient to the ED was significantly longer than the patients with accidental poisoning. Furthermore, the time to arrive at the ED was also associated with higher mortality among the patients. Out of the 41 patients who died after consuming poison intentionally, 10 (24.3%) presented to the ED after 180 minutes of poison exposure. A study reported that older age was associated with poor patient prognosis and increased risk of fulminant liver failure and death. Older people with late presentation also had a poorer prognosis than younger patients, with early presentation after paracetamol poisoning [13]. Similar findings have been reported locally where late presentation increased the risk of mortality among patients with a history of self-poisoning with organophosphates [14-15].

It should also be noted that older patients presented with severe symptoms compared to younger patients, which indicates the association between increasing age and worse patient prognosis [15-17].

A retrospective data of 22 years from Aga Khan Hospital revealed that the prevalence of poisoning was 0.3%. The majority of these cases, i.e., 65.5%, were adults, whereas 243 (34.5%) were younger than 16 years. More than three-fourths of the cases were in critical condition. The most common causes of poisoning were psychiatric drugs (647/705 [92%]) followed by prescription medications (172 [24.4%]), pesticides (108 [15.3%]), and hydrocarbons (71 [10%]). In a minority of the cases, the common causes were analgesics, household toxins, alcohol, drug abuse, and other poisonous substances [7].

One of the most critical factors contributing to the increased risk of suicide attempts is the easy access to drugs. For instance, benzodiazepines are readily available from pharmacies without a vigilant check on prescription. Moreover, these drugs are also very cheap, with the average price of a 30 tablet pack of 5 milligrams of diazepam costing only 16 Rupees [11].

In short, the time of ingestion of poison or exposure to arrival at the ED holds immense significance and directly impacts the patients' survival.

Prompt diagnosis and proper presenting history are two critical factors to a better patient outcome. It would further help increase the general practitioner's awareness level regarding different signs and symptoms of toxic exposure to establish a prompt diagnosis.

## Conclusions

Our study highlights some variations in accidental versus DSP cases. Women more often attempted suicide, whereas males suffered accidental poisoning more frequently. Firstly, we found a female predominance in the DSP group, whereas there was a male dominance in young children experiencing accidental poisoning. Longer time from ingestion of poison to the arrival is associated with poor patient prognosis.
